# Synthesis of neural networks for spatio-temporal spike pattern recognition and processing

**DOI:** 10.3389/fnins.2013.00153

**Published:** 2013-08-30

**Authors:** Jonathan C. Tapson, Greg K. Cohen, Saeed Afshar, Klaus M. Stiefel, Yossi Buskila, Runchun Mark Wang, Tara J. Hamilton, André van Schaik

**Affiliations:** The MARCS Institute, University of Western SydneyKingswood, NSW, Australia

**Keywords:** pseudoinverse solution, spatio-temporal spike pattern recognition, spiking network synthesis, kernel method, spike-time encoded information

## Abstract

The advent of large scale neural computational platforms has highlighted the lack of algorithms for synthesis of neural structures to perform predefined cognitive tasks. The Neural Engineering Framework (NEF) offers one such synthesis, but it is most effective for a spike rate representation of neural information, and it requires a large number of neurons to implement simple functions. We describe a neural network synthesis method that generates synaptic connectivity for neurons which process time-encoded neural signals, and which makes very sparse use of neurons. The method allows the user to specify—arbitrarily—neuronal characteristics such as axonal and dendritic delays, and synaptic transfer functions, and then solves for the optimal input-output relationship using computed dendritic weights. The method may be used for batch or online learning and has an extremely fast optimization process. We demonstrate its use in generating a network to recognize speech which is sparsely encoded as spike times.

## Introduction

There has been significant research over the past two decades to develop hardware platforms which are optimized for spiking neural computation. These platforms range from analog VLSI systems in which neurons are directly simulated by using CMOS transistors as ion channels and synapses, to highly parallel custom silicon microprocessor arrays (Boahen, 2006; Khan et al., 2008; Schemmel et al., 2010). Some of these platforms are now capable of modeling populations of over a million neurons, at rates which are significantly faster than biological real time.

The advent of these systems has revealed a lack of concomitant progress in algorithmic development, and particularly in the synthesis of spiking neural networks. While there are a number of canonical structures, such as Winner-Take-All (WTA) networks (Indiveri, 2001), and some spiking visual processing structures such as Gabor filter networks and convolutional neural networks are routinely implemented (Zamarreño-Ramos et al., 2013), there are few successful methods for direct synthesis of networks to perform any arbitrary task which may be defined in terms of spike inputs and spike outputs, or in terms of a functional input-output relationship.

One successful method is in the core algorithm for the Neural Engineering Framework (NEF; Eliasmith and Anderson, 2003). The NEF was first described in 2003, and generally builds large systems from subnetworks with a standard three-layer neural structure, in which the first layer are inputs; the second layer is a very large hidden layer of non-linear interneurons, which may have recurrent connections; and the third layer is the output layer, which consists of neurons with linear input-output characteristics. The connections between the input and hidden layers are randomly weighted, and fixed (they are not altered during training). The connections between the hidden and output layers are trained in a single pass, by mathematical computation rather than incremental learning. We will describe this structure in more detail in the following section.

The NEF core algorithm was perhaps the first example of a larger class of networks which have been named LSHDI networks—Linear Solutions of Higher Dimensional Interlayers (Tapson and van Schaik, 2013). These are now widely used in the machine learning community in the form of the Extreme Learning Machine (ELM; Huang et al., 2006a)—a conventional numerical neural network, which performs with similar accuracy to Support Vector Machines (SVMs) and which is significantly quicker to train than SVMs. Both the ELM and NEF methods have been applied to implement bio-inspired networks on neural computation hardware (Choudhary et al., 2012; Conradt et al., 2012; Galluppi et al., 2012; Basu et al., 2013). Most recently, Eliasmith and colleagues have used the method to synthesize subnetworks in a 2.5 million neuron simulation of the brain (Eliasmith et al., 2012). This illustrates that the NEF is a meta-level framework for building cognitive systems, in which the LSHDI networks that are referred to in this report form only the building blocks.

The NEF is an effective synthesis method, with three important caveats: it intrinsically uses a spike rate-encoded information paradigm; it requires a very large number of neurons for fairly simple functions (for example, it is not unusual for a function with two inputs and one output, to use an interlayer of fifty to a hundred spiking neurons); and the synthesis (training) of weights is by mathematical computation using a singular value decomposition (SVD), rather than by any biologically plausible learning process.

We have recently addressed the third of these caveats by introducing weight synthesis in LSHDI through an online, biologically plausible learning method called OPIUM—the Online PseudoInverse Update Method (Tapson and van Schaik, 2013). This method also allows for adaptive learning, so that if the underlying function of the network changes, the weights can adapt to the new function.

The relative merits of rate-encoding and time- or place-encoding of neural information is a subject of frequent and ongoing debate. There are strong arguments and evidence that the mammalian neural system uses spatio-temporal coding in at least some of its systems (Van Rullen and Thorpe, 2001; Masuda and Aihara, 2003), and that this may have significant benefits in reducing energy use (Levy and Baxter, 1996). A synthesis method which can produce networks for temporally encoded spike information will have significant benefits in terms of modeling these biological systems, and in reducing the quantity of spikes used for any given information transmission.

In this report we describe a new neural synthesis algorithm which uses the LSHDI principle to produce neurons that can implement spatio-temporal spike pattern recognition and processing; that is to say, these neurons are synthesized to respond to a particular spatio-temporal pattern of input spikes from single or multiple sources, with a particular pattern of output spikes. It is thus a method which intrinsically processes spike-time-encoded information. The synthesis method makes use of multiple synapses to create the required higher dimensionality, allowing for extreme parsimony in neurons. In most cases, the networks consist only of input neurons and output neurons, with the conventional hidden layer being replaced by synaptic connections. These simple networks can be cascaded to perform more complex functions. The starting point of the synthesis method is to have an ensemble of input channels emitting neuron spike trains; these are the input neurons. The desired output spike trains are emitted by the output neurons, and our method is used to generate the synaptic connectivity that produces the correct input-output relationship. We call this method the Synaptic Kernel Inverse Method (SKIM). Training may be carried out by pseudoinverse method or any similar convex optimization, so may be online, adaptable, and biologically plausible.

The point of departure between this new method and our prior work (Tapson and van Schaik, 2013) is that the prior work (OPIUM) was suitable for solving conventional LSHDI problems but made no particular contribution to the special case of spike-timing dependent signals. The work described here is specifically aimed to provide a synthesis method for systems in which perhaps only a single spike, or none at all, is received in each channel during an observation interval. Methods which work on rate-based spike signals are generally dysfunctional in this regime, but it is considered to be widely used in mammalian neural signaling.

This work also offers a synthesis method for networks to perform cortical sensory integration as postulated by Hopfield and Brody (2000, 2001). This required that short, sparse spatio-temporal patterns be integrated to produce recognition of a learned input. In section Results below, we show a detailed methodology for solving Hopfield and Brody's *mus silicium* challenge with the SKIM method.

There are a number of published network methodologies which process spatio-temporal spike patterns. These include reservoir computing techniques such as liquid state machines (Maass et al., 2002; Maass and Markram, 2004) and echo state networks (Jaeger, 2003). In particular, Maass and colleagues have analyzed the requirements for universal computation in terms of networks of these types, and have identified requirements such as network stability, input separability and fading memory as being necessary conditions (Maass and Sontag, 2000). We will refer to this work in more detail in section Methods.

An interesting feedforward network for spatio-temporal pattern recognition is the Tempotron of Gütig and Sompolinksy (2006). The Tempotron consists of a leaky integrate-and-fire neuron with a number of synaptic inputs. The synaptic weights are trained by gradient descent so that the neuron exceeds its threshold for particular input patterns of spikes. A similar system, the DELTRON, has been implemented on an FPGA platform using neuromorphic principles (Hussein et al., 2012). The Tempotron and DELTRON are two special cases of the type of network which can be synthesized using the methodology outlined in this report. Gütig and Sompolinksy have subsequently extended the Tempotron concept with adaptive shunting inhibition at the synapses, which produces a very impressive robustness to time-warping in the system's performance in speech recognition (Gütig and Sompolinksy, 2009). This principle may well be implementable in most spatio-temporal pattern recognition networks, including that which is reported on here.

A feature of the Tempotron is that the weights are learned incrementally, rather than synthesized. This report focuses on a synthesis method for networks; that is to say, one in which the network or synaptic weights are calculated analytically, rather than learned. The advantage of synthetic methods are in speed of development, and also in robustness of outcomes, as learning methods tend to be intrinsically stochastic and solutions are not necessarily repeatable. Nonetheless, it has been shown that learning methods such as spike-timing dependent plasticity (STDP) can produce extremely sensitive spatio-temporal pattern recognition (Masquelier et al., 2006, 2009). There are also hybrid methods in which combinations of synthesis and evolution have been used to find the parameters for network weights and neurons (Russell et al., 2010; Torben-Nielsen and Stiefel, 2010). More recently, learning methods such as ReSuMe (Remotely Supervised Method, by Ponulak and Kasiñski, 2010) and SPAN (Mohemmed et al., 2012) have been proposed as variations on the classic Widrow–Hoff or Delta rule.

## Methods

### The LSHDI principle

**Figure 1:**
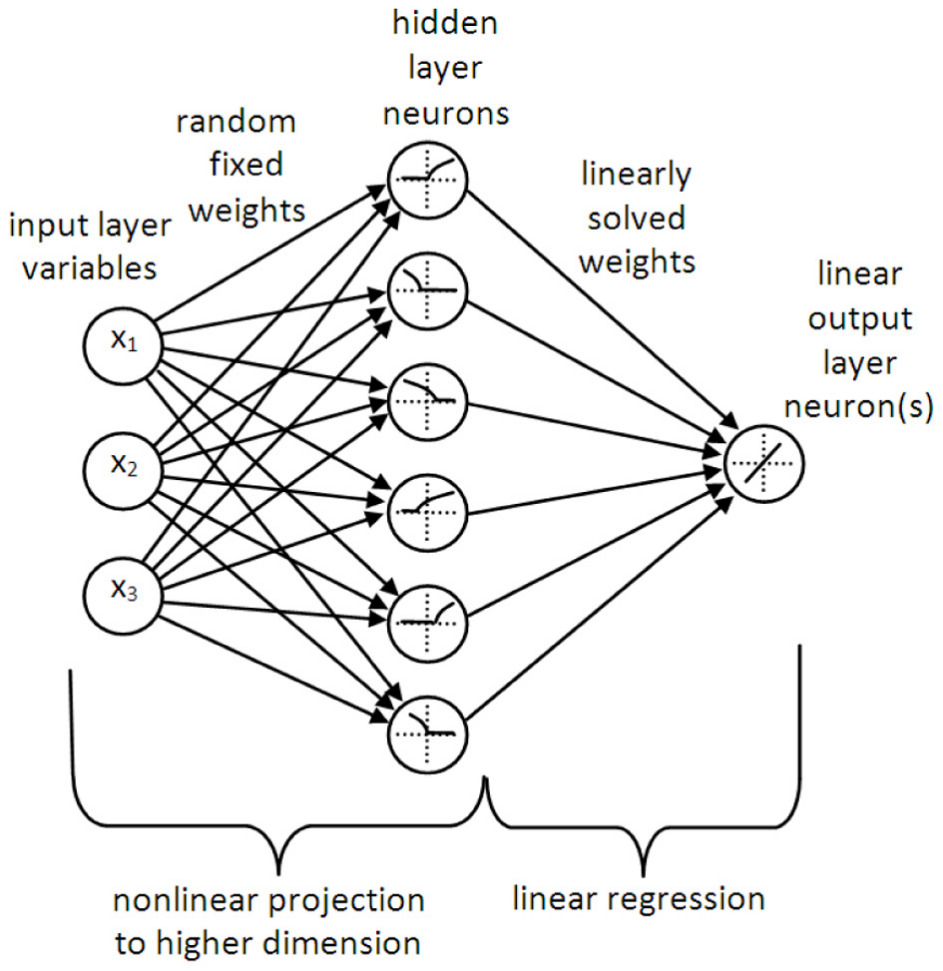
**A typical LSHDI network**. The input variables are projected to a higher dimension (in this case, from 3D to 6D) by means of random fixed weights and a non-linear transformation (which in the case of NEF may be a leaky integrate-and-fire neuron, as inferred here). The outputs from the higher dimensional space are weighted and summed by linear output neurons, allowing for solution of the output weights by linear regression or classification.

LSHDI networks are generally represented as having three layers of neurons—the classic input, hidden and outer layer feedforward structure (see Figure 1). Should a memory function be desired, the hidden layer may have recurrent connections. However, LSHDI networks differ from regular feedforward networks in three important respects. The hidden layer is usually much larger than the input layer (values of 10–50 times are used by various practitioners; Huang and colleagues have tested networks in which the number is incrementally increased—Huang et al., 2006a; Huang and Chen, 2008). The connections from the input layer to the hidden layer are randomly generated, and are not changed during training. Finally, the output layer neurons have a linear response to their inputs.

The key to the success of LSHDI networks is that they make use of the non-linear transformation that lies at the core of kernel methods such as kernel ridge regression and SVMs. This is a process by which data points or classes which are not linearly separable in their current space, are projected non-linearly into a higher dimensional space (this assumes a classification task). If the projection is successful, the data are linearly separable in the higher dimensional space. In the case of regression or function approximation tasks, the problem of finding a non-linear relationship in the original space is transformed into the much simpler problem of finding a linear relationship in the higher dimensional space, i.e., it becomes a linear regression problem; hence the name Linear Solutions of Higher Dimensional Interlayers.

A number of researchers have shown that random non-linear projections into the higher dimensional space work remarkably well (Rahimi and Recht, 2009; Saxe et al., 2011). The NEF and ELM methods create randomly initialized static weights to connect the input layer to the hidden layer, and then use non-linear neurons in the hidden layer (which in the case of NEF are usually leaky integrate-and-fire neurons, with a high degree of variability in their population). Many other projection options have also been successful, perhaps summed up by the title of Rahimi and Recht's paper, “Weighted Sums of Random Kitchen Sinks” (Rahimi and Recht, 2009). This paper is recommended to the reader both for its admirable readability, and the clarity with which it explains the use of random projection as a viable alternative to learning in networks. As shown by Rahimi and Recht, random non-linear kernels can achieve the same results as random weighting of inputs to non-linear neurons.

The linear output layer allows for easy solution of the hidden-to-output layer weights; in NEF this is computed in a single step by pseudoinversion, using SVD. In principle, any least-squares optimal regression method would work, including, for example, linear regression. We note that for a single-layer linear regression solution such as this, the problem of getting trapped in a local minimum when using gradient descent optimization should not occur, as the mapping is affine and hence this is a convex optimization problem.

The LSHDI method has the advantages of being simple, accurate, fast to train, and almost parameter-free—the only real decisions are the number of interlayer neurons and the selection of a non-linearity, and neither of these decisions is likely to be particularly sensitive. A number of studies have shown that ELM implementations remain stable and have increasing accuracy as the number of interlayer neurons is increased (Huang et al., 2006b; Huang and Chen, 2008); however, this robustness has not been proven in theory.

### LSHDI for spike time encoded neural representations—the SKIM method

Spike time encoding presents difficulties for conventional neural network structures. It is intrinsically event-based and discrete rather than continuous, so networks based on smoothly continuous variables do not adapt well into this domain. Outside of simple coincidence detection, it requires the representation of time and spike history in memory (the network must remember the times and places of past spikes). The output of the network is also an event (spike) or set of events, and therefore does not map well to a linear solution space.

**Figure 2:**
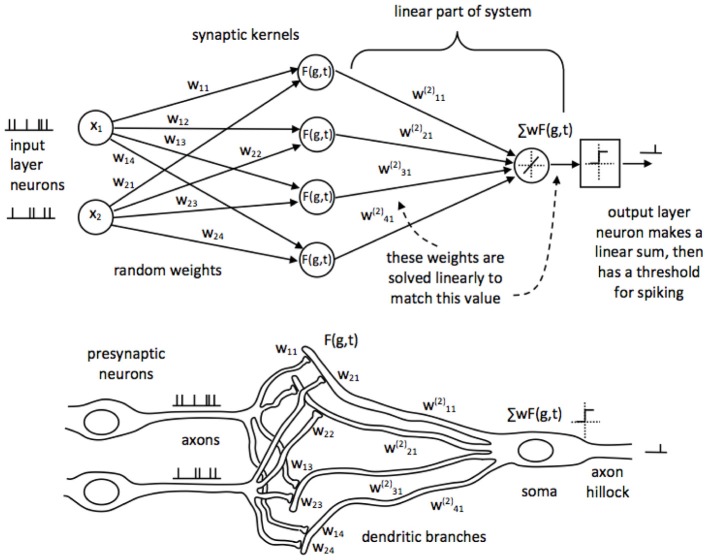
**The SKIM network structure for time-encoded spike processing, shown in LSHDI and biological form**. Presynaptic neurons are connected to a postsynaptic neuron through randomly generated, fixed weighted synapses. Synapses are implemented as filter elements which produce a non-linear impulse response, in response to incoming spikes. The postsynaptic dendritic branch acts as a hidden layer element, and integrates the synaptic currents by means of a non-linear time-persistent filter. Memory may be implemented specifically as axonal or dendritic delays, or in terms of axonal functions. Dendritic signals are summed at the soma, and if they exceed a threshold, the axon hillock emits a spike.

We have developed a biologically plausible network synthesis method in which these problems are addressed. The basic network consists of presynaptic spiking neurons which connect to a spiking output neuron, via synaptic connections to its dendritic branches, as illustrated in Figure 2. The synapses are initialized with random weights which do not change thereafter; this, together with a subsequent non-linearity, provides the projection to a higher dimension required for the improved separability. The dendritic branches sum the synaptic input currents. Some user-selected feature of the network—recurrent connections, axonal or dendritic delay, synaptic functions, or some combination of these—implements memory (in the form of persistence of recent spikes); and there must be a non-linear response, which provides the non-linearity in projection necessary for improved separability. In the top schematic in Figure 2 we have renamed the hidden layer as synapses, to emphasize that these (the hidden layer elements) are not spiking neurons.

The outputs from the dendritic branches are summed in the soma of the output neuron. At this stage we are able to use a linear solution to calculate the correct weights for the connection between dendritic branches and soma; solution by pseudoinverse or backpropagation will both work.

The linear solution solves the dendritic weights required to produce soma values which are below threshold for non-spike times and above threshold for spike times. The soma potential value for which the linear weights are calculated can be set to be one of two binary values, as in a classifier output; for example, it can be set to unity at spike output times, and zero when no spike is wanted. This may not be necessary in some applications where an analog soma potential would be a useful output. The final output stage of the neuron is a comparator with a threshold for the soma value, set at some level between the spike and no-spike output values. If the soma potential rises above the threshold, a spike is generated; and if it does not, there is no spike. This represents the generation of an action potential at the axon hillock.

The reason that this network works is that it converts discrete input events into continuous-valued signals within the dendritic tree, complete with memory (the synapses and dendritic branches may be thought of as infinite-impulse response filters); and at the same time this current and historic record of input signals is projected non-linearly into a higher-dimensional space. The spatio-temporal series of spikes are translated into instantaneous membrane potentials. We can then solve the linear relationship between the dendritic membrane potentials and the soma potential, as though it was a time-independent classification problem: given the current membrane state, should the output neuron spike or not? The linear solution is then fed to the comparator to generate an event at the axon of the output neuron.

One issue is that when output spikes are sparse (which is a common situation) there is little impetus for the network to learn non-zero outputs. We have increased the quality of learning by adding non-zero weight to the target sequences, by increasing either the target output spike amplitude, or width, or both. In most cases it is more appropriate to increase the width (as in the example network of section 3.2, in which the exact timing of outputs is not explicitly available anyway). It is also often the case that the optimum output threshold is not half of the spike amplitude, as might be expected; we have found as a guideline that a threshold of 25% of spike amplitude is more accurate, which reflects this problem to some extent.

The inputs to this method do not necessarily need to be spikes. The method will work to respond to any spatio-temporal signals which fall within an appropriate range of magnitude. However, given that the target for this work is synthesis of spatio-temporal spike pattern processing systems, we analyze the system for spiking inputs.

### Synaptic kernels

In the SKIM method, the hidden layer synaptic structure performs three functions:

1. The axon signals are weighted and transmitted to the dendritic branch, which sums inputs from several axons.
2. The axon signals are non-linearly transformed. This is necessary to ensure the non-linear projection to a higher dimension; a linear projection would not improve the separability of the signals.
3. The axon signals are integrated, or otherwise transformed from Dirac impulses into continuous signals which persist in time, in order to provide some memory of prior spike events. For example, the use of an alpha function or damped resonance to describe the synaptic transfer of current, as is common in computational neuroscience, converts the spikes into continuous time signals with an infinite impulse response.

The sum of these transformed signals represents the proximal dendritic response to its synaptic input.

As mentioned in the previous section, steps 1, 2, and 3 may be re-ordered, given that step 3 is most likely to be linear. Any two of the steps may be combined into a single function (for example, integrating the summed inputs using an integrator with a non-linear leak).

We refer to the hidden layer neuron structure that performs steps 1–3 above as the *synaptic kernel*. It is generally defined by the synaptic or postsynaptic function used to provide persistence of spikes, and this may be selected according to the operational or biological requirements of the synthesis. We have used leaky integration, non-linear leaky integration, alpha functions, resonant dendrites, and alpha functions with fixed axonal or dendritic delays; all of which work to a greater or lesser extent, if their decay time is of a similar order of magnitude to the length of time for which prior spikes must be remembered. Linear leaky integration is equivalent to a neuron with recurrent self-connection (with gain chosen to ensure stability). We characterized this as an infinite-impulse response filter earlier, but we note that it may be reasonable to truncate the spike response in time (or use a finite-response function) to ensure stability.

Maass and Sontag (2000) identified the requirements for network stability, fading memory and pointwise separation as necessary for universal computation in recurrently connected networks. In avoiding recurrent connections we have ensured stability, and the use of synaptic kernels with non-linear compression provides fading memory in a way very similar to that suggested by Maass and Sontag; they also note that “A biologically quite interesting class of filters that satisfies the formal requirement of the pointwise separation is the class of filters defined by standard models for dynamic synapses.” The non-linear compression following a linear synaptic filter has the effect of giving the whole synapse depressive adaptation, as a second spike arriving at the synapse soon after a first spike will be compressed to a higher degree, given the filter output is not starting from zero for the second spike.

Table 1 shows some typical synaptic functions in mathematical and graphical form.

**Table 1:** **Typical synaptic kernels in mathematical and graphical form**.

**Kernel type**	**Mathematical expression for filter response**	**Typical function (Spike at *t* = 0)**
Stable recurrent connection (leaky integration) with non-linear leak	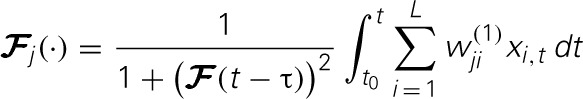	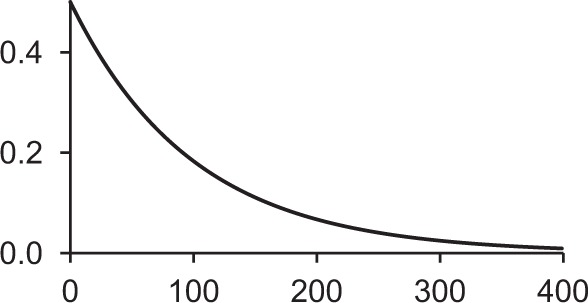
Alpha function followed by compressive non-linearity	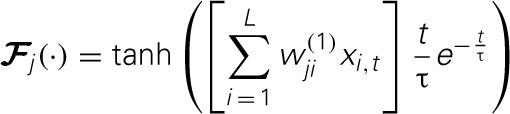	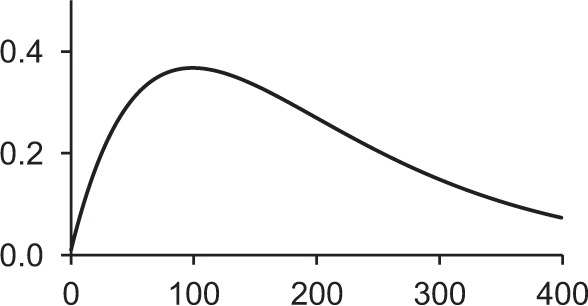
Damped resonant synapse followed by compressive non-linearity	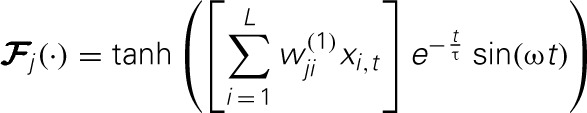	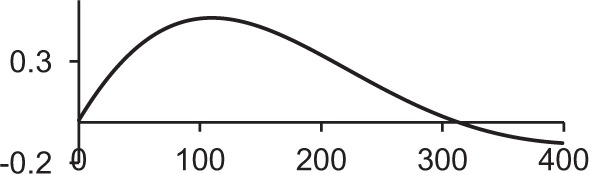
Synaptic or dendritic delay with alpha function, followed by compressive non-linearity	*for t ≥ △T:* 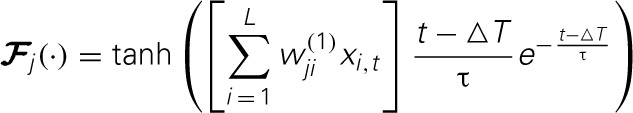	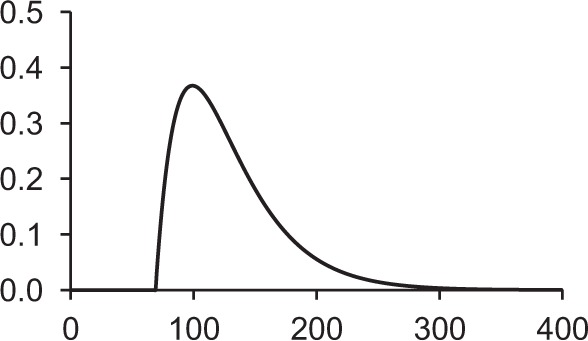
	*for t < △T:* 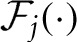 = 0	
Synaptic or dendritic delay with Gaussian function, followed by compressive non-linearity	*for t ≥ △T*: 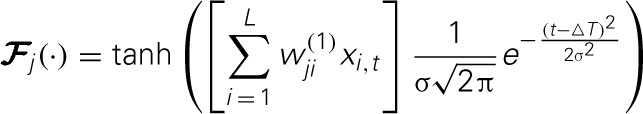	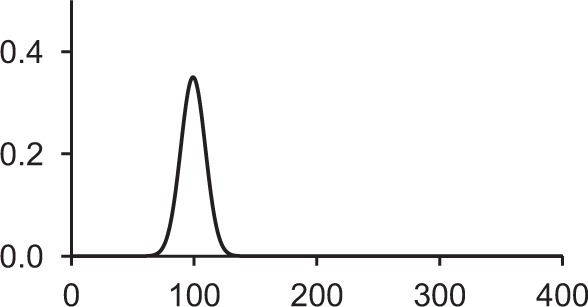
	*for t < △T:* 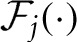 = 0	

The order from top to bottom provides increasingly precise delay timing; with the exception of the leaky integrator, time constants were chosen for maximum synaptic transmission at 100 timesteps after spiking. Variables are as for Equation 1. τ is the time constant for the various functions, ΔT is an explicit synaptic or dendritic delay, and ω the natural resonant frequency for a damped resonant synaptic function. The horizontal axes indicate timesteps.

The synaptic kernels perform a similar synthetic function to wavelets in wavelet synthesis. By randomly distributing the time constants or time delays of the functions, a number of different (albeit not necessarily orthogonal) basis functions are created, from which the output spike train can be synthesized by linear solution to a threshold. An analogous process is spectral analysis by linear regression, in which the frequency components of a signal, which may not necessarily be orthogonal Fourier series basis functions, are determined by least-squares error minimization (Kay and Marple, 1981).

We may address the issue of resetting (hyperpolarizing) the soma potential after firing an output spike. This is simple to implement algorithmically (one can simply force all the dendritic potentials to zero after a firing event) and may improve the accuracy; our experiments with this have not shown a significant effect, but it may be present in other applications.

### Analysis of the SKIM method

The SKIM method may be implemented using a number of different synaptic kernels, but we can outline the method for a typical implementation. The inputs may be expressed as an ensemble of signals ${\bar{x}}_{t}$ ∈ ℝ^*L* × 1^ where *t* is a time or series index, and each element of *x* represents the output of a presynaptic neuron. For convenience, the signal magnitudes may take values *x*_*i*_ ∈ {0, 1} depending on whether there is a spike from neuron *i* at time *t* or not. The signals propagate from the presynaptic axons to synaptic junctions with the dendritic branches of the postsynaptic neuron (for the sake of clarity, we will restrict the output to a single postsynaptic neuron at this stage; note that each dendritic branch has different characteristics, and hence has a unique index *j*). The synaptic weights *w*^(1)^_*ji*_are fixed to random values (we can postulate a uniform distribution in some sensible range, although Rahimi and Recht (2009) have shown that this is not necessary). The superscript indicates the weights' layer. The dendrites and output neuron process the incoming signals as follows: 
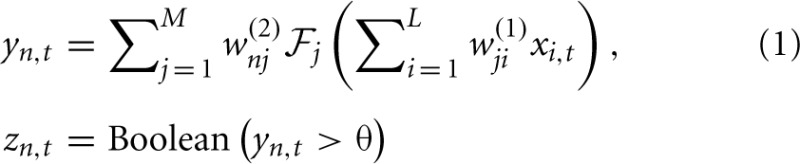


where 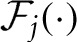 is a non-linear filter function operating on the weighted and summed input spikes; we may use different functions for different dendrites, hence the subscript. Note that most filters will consist of a linear function and a non-linearity; the non-linear function and the integral may be swapped in order, if that better represents the required neural functionality, but there is a significant loss of computational power if the non-linearity precedes the filter function, as the non-linearity is then acting on scaled delta functions rather than continuous-time signals, so each event is independently scaled—with continuous signals, it is the sum of synaptic responses that is non-linearly scaled, giving effects of adaption according to spike history. The LSHDI method obtains a result in either case, but much of the computational power identified by Maass and Sontag (2000) is lost. Here *y*_*t*_ ∈ ℝ^*N* × 1^ is the output from the linear soma element, prior to thresholding; the output after comparison with threshold θ is *z*_*t*_ ∈ {0, 1}^*N* × 1^ (spikes or no spikes). Each soma element *y*_*n,t*_ is a linear sum of the *M* hidden layer dendritic outputs weighted by *w*^(2)^_*nj*_. *n* is the output (soma) vector index, *j* the hidden layer (dendritic) index, and *i* the input (neuron) vector index. The dendritic outputs depend on the dendrite's filter function 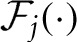 and the randomly determined synaptic weights *w*^(1)^_*ji*_between input and hidden layer.

As mentioned previously, the synaptic weights *w*^(1)^_*ji*_ are randomly set (usually with a uniform distribution in some appropriate range) and remain fixed. The training of the network consists of calculating the weights *w*^(2)^_*nj*_ connecting the dendrites to the soma. This is performed in a single step (for a batch learning process) using a linear regression solution. If we define the dendrite potentials at the synapses to be 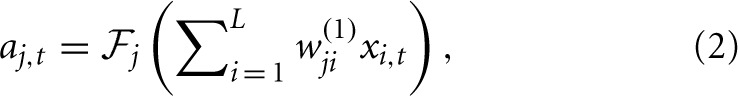


we can represent the outputs *a* of the hidden layer in the form of a matrix *A* in which each column contains the hidden layer output for one sample in the time series, with the last column containing the most recent sample; *A* = [*a*_1_ … *a*_*k*_] where *A* ∈ ℝ^*M* × *k*^. Similarly we can construct a matrix *Z* of the corresponding output values; *Z* = [*z*_1_ … *z*_*k*_] where *Z* ∈ {0, 1}^*N* × *k*^. Note that *Z* is expected to consist of binary or Boolean values; spike or non-spike. Synthesizing the network requires that we find the set of weights *W* ∈ ℝ^*N* × *M*^that will minimize the error in:

(3) WA = Z.

This may be solved analytically by taking the Moore-Penrose pseudoinverse *A*^+^ ∈ ℝ^*k* × *M*^ of *A*:

(4) $$W=ZA^{+}.$$

In a batch process, *A* and *Z* will be static data sets and the solution can be obtained by means of SVD. In a recent report (Tapson and van Schaik, 2013), we have described an incremental method for solving the pseudoinverse, which we called OPIUM—the Online PseudoInverse Update Method. OPIUM may be used for online learning, or where batch data sets are so large that the matrix sizes required for the SVD are too unwieldy in terms of computational power and memory.

The synaptic kernel may be selected according to the operational or biological requirements of the synthesis; for example, if exact timing is critical, an explicit delay with narrow Gaussian function may produce the best results, but if biological realism is most important, an alpha function might be used. We have used a number of mathematically definable non-linearities, but there is no reason why others, including arbitrary functions that may be specified by means of e.g., a lookup table, could not be used. There is no requirement of monotonicity, and we have successfully used wavelet kernels such as the Daubechies function, which are not monotonic.

We note that synaptic input weights may be both positive and negative for the same neuron, which would not be biologically realistic. In practice, we could limit them to one polarity simply by limiting the range of the random distribution of weights. This would produce networks which would in most cases be less versatile, but the opportunity exists to combine excitatory and inhibitory networks in cases where biological verisimilitude is a high priority.

## Results

### An example of the SKIM method

Consider a situation in which we wish to synthesize a spiking neural network that has inputs from five presynaptic neurons, and emits a spike when, and only when, a particular spatio-temporal pattern of spikes is produced by the presynaptic neurons. We create an output neuron with 100 dendritic branches, and make a single synapse between each presynaptic neuron and each dendritic branch, for a total of 500 synapses. (This gives a “fan-out” factor of 20 dendrites per input neuron, which is an arbitrary starting point; we will discuss some strategies for reducing synapse and dendrite numbers, should synaptic parsimony be a goal). The structure is therefore five input neurons, each making one synapse to each of 100 dendritic branches of a single output neuron.

**Figure 3:**
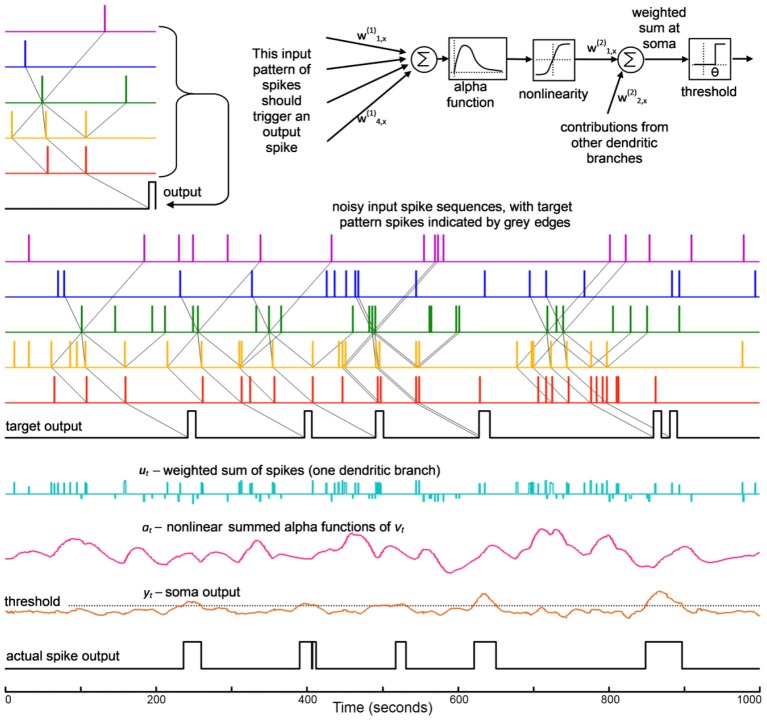
**This shows the development of the SKIM method for a spatio-temporal spike pattern recognition system**. The structure of one dendritic branch and the soma is shown at the top. The signals are, from top: the input pattern on five channels and the target output; a test sequence with added noise spikes, and target output; the summed spike output from one dendritic branch; the resulting non-linearly transformed alpha function output from that branch; the soma potential for the output neuron; and the resulting output spike train. It can be seen that the spike pattern is successfully recognized in the presence of some spike noise. The target output sequence has spikes which have been widened to 10 timesteps in order to increase the non-zero target energy, as described in the text. Note that where input patterns overlap closely, the output spikes may merge together and appear as one event, as seen at 630 and 850 ms.

The pattern to be detected consists of nine spikes, one to three from each neuron, separated by specific delays. This pattern will be hidden within random “noise” spikes (implemented with a Poisson distribution)—see Figure 3.

In this example, we use the following functions for summing, non-linearity, and persistence. A summed signal *u*_*j,t*_ is obtained conventionally:

(5) $$u_{j,t}=\sum_{i=1}^{L}w_{ji}^{\left(1\right)}x_{i,t}$$

Note that *L* = 5 in this example, and the weights *w*^(1)^_*ji*_are randomly (uniformly) distributed in the range (−0.5, 0.5). After summing, the synaptic function is used to provide persistence of the signal in time, as follows: at any timestep *t*_0_, if *u*_*j, t*_0__ ≠ 0 (i.e., there is a spike), a function *v*_*j,t*_ (*u*_*j, t*_0__), *t* > *t*_0_, is added to the dendritic branch signal *a*_*j,t*_. In this example *v*_*j,t*_ is an alpha function scaled by the amplitude of *u*_*j,t*_0__ and with its origin at *t*_0_:

(6) $$v_{j,t}=u_{j,t_{0}}\left(\frac{t-t_{0}}{t_{s}}e^{-\frac{t-t_{0}}{t_{s}}}\right)$$

The time constant *t*_*s*_ of the alpha function will define the persistence of the spike input in time. In practice we have found *t*_*s*_ ≈ *t*_max_/2 to be a useful heuristic, where *t*_max_ is the longest time interval for which spikes will need to be “remembered.” In this case, the maximum length of the pattern was 200 timesteps, and the values of *t*_*s*_ were uniformly distributed in the range (0, 100) timesteps, thereby straddling the heuristic value. This heuristic applies only to the alpha function; other kernels will require some random distribution of time constants or delays in some similarly sensible range.

Note that the length of time for which sustained non-zero power is maintained in the impulse response of the synaptic kernels defines the length of memory in the network, and the point of maximum amplitude in the impulse (spike) response of a kernel filter gives a preferred delay for that particular neural pathway.

The logistical function is used to non-linearly transform the summed values:

(7) $$a_{j,t}=\frac{1}{1+e^{-kv_{j,t}}}-0.5$$

Here *k* = 5 is a scaling constant.

Figure 3 shows the development of the signals through the system.

The network was presented with a mixture of Poisson-distributed random spikes and Poisson-distributed spike patterns, such that the number of random noise spikes was approximately equal to the number of pattern spikes. Pattern spikes were exact copies of the original pattern; however the broad peaks of the synaptic kernels have the effect of producing broad somatic responses, as are visible in the synaptic responses and soma signals shown in Figure 3, which create some tolerance for spike jitter. An output spike train was used to provide the solution data during the training calculation (note that while input events were effectively instantaneous, taking just one time step, the output train consisted of slightly wider windows of ten time steps, on the basis that a spike within some short window would constitute a functional output; the output spike is also displaced moderately later in time than the last input spike, in order to allow for the dendritic response to peak. In summary, the target spike consisted of a square pulse of unitary amplitude and ten timesteps in length, displaced ten timesteps after the end of the input sequence). The broader target “spike” causes the network to train for a broad output spike, the effect of which may be seen more clearly in Figure 5. The results for the present synthesis can be seen in Figure 3. This shows the performance of a network which has been trained with ~580 presentations of the target in a sequence 10^5^ timesteps in length (the target occurs in a nominal 200-timestep window and there was frequently an overlap of patterns, as can be seen in the figure). Target patterns may contain zero, single or multiple spikes per input channel.

In examining the issue of resetting the somatic potential, we note that it is generally accepted that the Markov property applies to integrate-and-fire or threshold-firing neurons (Tapson et al., 2009); so that the dependence of the firing moment of a neuron is not dependent on the history of the neuron prior to the most recent spike. It might seem intuitively necessary that this only holds if the neuron is reset to a potential of zero (hyperpolarized) after the most recent spike, but in fact from the point of view of the trajectory of the membrane potential, and the inverse solution of the dendritic weights needed to produce that trajectory, it is immaterial what the potential starting level is, as long as it is defined and consistent. Those who are concerned by this issue may cause their simulation code to reset the membrane potential after spiking.

### Use of the SKIM method on a predefined problem

In this section we will illustrate the use of the SKIM method to solve a problem in spatio-temporal pattern recognition. In 2001, John Hopfield and Carlos Brody proposed a competition around the concept of short-term sensory integration (Hopfield and Brody, 2000, 2001). Their purpose was to illustrate the usefulness of small networks of laterally- and recurrently-connected neurons, and part of the competition was to develop a network to identify words based on a very sparse representation of audio data. The words were spoken digits drawn from the TI46 corpus (TI46, 2013), and were processed in a quasi-biological way; they were passed through a cochlea-like filterbank to produce 20 parallel narrowband signals, and then the times of onset, offset, and peak power were encoded as single spikes at that time, in separate channels; so, each word was encoded as an ensemble of single spikes on multiple channels.

Hopfield and Brody's neural solution—referred to as *mus silicium*, a mythical silicon-based mouse-like lifeform—was based on neurons which exhibited bursting spiking, with a linearly decaying time response, to input spikes. The key to its operation was that this linear decay offered a linear conversion of time to membrane potential amplitude, and thereby the encoding of time, which then enabled the recognition of spatio-temporal patterns; and that coincidence of signal levels could be detected by synchronized output spiking. In the SKIM method, we achieve a similar result (without bursting spikes), using synapses with arbitrary time responses, which allows a significantly greater degree of biological realism together with sparser use of neurons and sparser use of spikes. It remains to be shown that the SKIM method is actually capable of solving the problem, and we outline its use for this purpose here.

Hopfield and Brody preprocessed the TI46 spoken digits to produce 40 channels with maximally sparse time encoding—a single spike, or no spike, per channel per utterance (a full set of onset, offset and peak for all 20 narrowband filters would require 60 channels, but Hopfield and Brody chose to extract a subset of events—onsets in 13 bands, peaks in 10 bands, and offsets in 17 bands). The spikes encode onset time, or peak energy time, or offset time for each utterance. Examples are shown in Figure 5.

Hopfield and Brody's original *mus silicium* network contained three or four layers of neurons, with an input layer (arguably two layers, as it spreads the input from 40 to 800 channels); a hidden layer with excitatory and inhibitory neurons, and significant numbers of lateral connections (75–200 synaptic connections each); and an output layer with one neuron per target pattern. The input layer was not encoded as one input per channel, but each channel was encoded with 20 different delays, to produce 800 input neurons. There were apparently 650 hidden layer neurons (a number of 800 is also referred to (Hopfield and Brody, 2001); elsewhere, *mus silicium* is described as having 1000 neurons in total (Wills, 2004, p. 4). The discrepancies may be due to whether input and output neurons were counted as well as hidden layer neurons, and whether non-functional neurons had been pruned). Input layer neurons were designed to output a burst of spikes for each input spike, so the original input pattern of 40 spikes would be scaled up to 800 series of 20–50 spikes each, as the inputs to the hidden layer.

By contrast, we will demonstrate the use of the SKIM method to produce a feedforward-only network with just two layers of neurons—40 input neurons (one per input channel) and 10 output neurons (one per target pattern). The presynaptic neurons will be connected by ten synapses each to each postsynaptic neuron, for a total of 400 synapses per postsynaptic (output) neuron. This gives the network a total of 50 spiking neurons connected by 4000 synapses.

The exact choice of synaptic kernel is not critical for success in this system. A simple α-function performs extremely well, as do synapses with a damped resonant response. In the data which follow, we show results for a number of different functions.

The prescribed training method for *mus silicium* was extremely stringent; it could be trained on only one single utterance of the target digit (“one”), interspersed with nine randomly selected utterances of other digits. The task was a real test of the ability of a network to generalize from a single case. In order to achieve the robustness to time-warping of the utterances for different speakers and different speech cadences, we produced a training set in which the exemplar pattern and its nine random companions were reproduced with a range of time warping from 76 to 124% of the originals.

Having been trained on this very small data set, the network is then tested on the full set of 500 utterances (which includes the examplar and nine random utterances, and therefore has 490 unseen utterances), almost all by previously unheard speakers.

### Results for the *mus silicium* problem

There are no published data for the accuracy of Hopfield and Brody's network, but the winning entry in their competition, from Sebastian Wills, is extensively described (Wills, 2001, 2004) and Hopfield and Brody's results are available online in archived copies of their website (Hopfield and Brody, 2013). The network was tested with 500 utterances of the digits 0–9, giving 50 target utterances of the digit “one” (only one of which was the exemplar) and 450 non-targets; and the error was defined as:

(8) $$\text{error}=\frac{\#\text{ false negatives}}{\#\text{ true positives}}+\frac{\#\text{ false positives}}{\#\text{ true negatives}}$$

**Figure 4:**
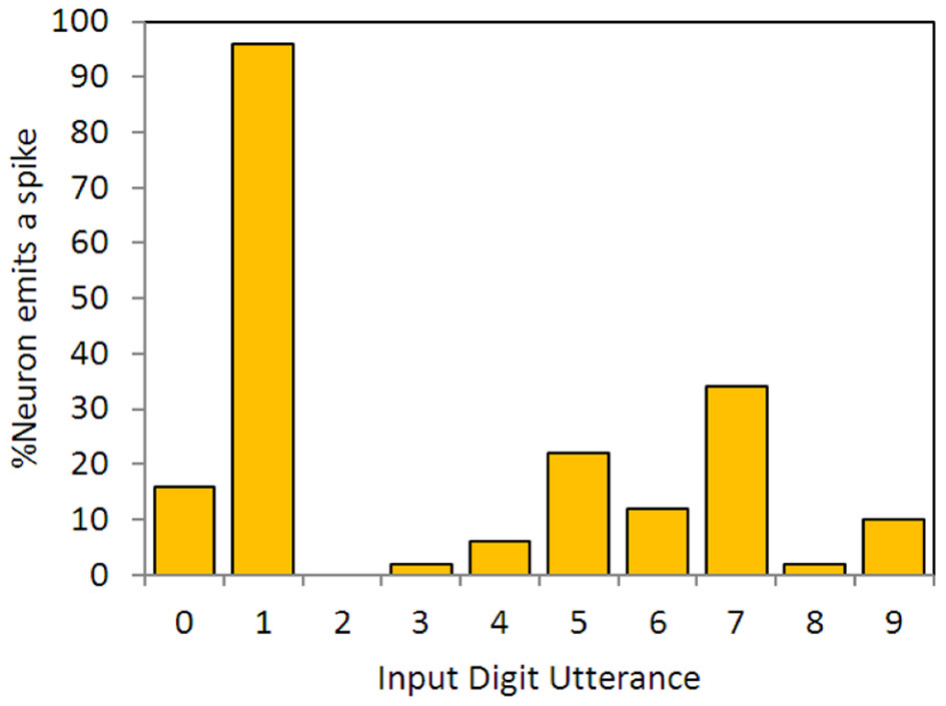
**Output neuron responses for a neuron synthesized to spike in response to the utterance of digit “one,”trained on a single exemplar**. The data show responses for the full set of 500 previously unseen time-encoded digit utterances, as in Hopfield and Brody (2000, 2001, 2013), Wills (2001, 2004).

Wills' minimum error was 0.253; Hopfield and Brody cite an error of 0.15. Errors smaller than this are easy to achieve with SKIM—see Table 2 below. Figure 4 shows performance on the test data set for the neuron trained on the single training utterance of “one,” and is the equivalent for a SKIM network to Wills' Figure 2.18 (Wills, 2004: p. 26). The test as defined only requires results for “one,” but as for Wills and Hopfield and Brody, we have constructed a network which can recognize all 10 digits.

**Table 2:** **Errors for SKIM networks applied to the *mus silicium* problem, with various different types of synaptic kernels**.

**Network**	**Error**
Wills, 2001	0.253
SKIM, Alpha synapse	0.224
SKIM, Damped resonance	0.183
SKIM, Delay plus alpha	0.173
SKIM, Delay plus Gaussian	0.169
Hopfield and Brody, 2013	0.15

All networks had the minimum 40 input neurons and 10 output neurons, and were connected with 40 × 10 × 10 = 4000 synapses in total.

The authors would like to make it clear that the results in Table 2 do not imply that this network would have won the *mus silicium* competition, as that competition had explicit restrictions on synaptic time constants that would have excluded a SKIM network; there was also a requirement for a test of robustness to weight change that is not practically applicable in a network with two layers of neurons (the competition assumed a three-layer network; or strictly speaking, four layers of neurons if the initial input spreading is taken into account). Nonetheless, we believe the SKIM performance on this problem illustrates its usefulness as a synthesis method for spatio-temporal pattern recognition.

Maass et al. (2002) also made use of the *mus silicium* data set in evaluating the liquid state machine, but unfortunately they modified the preprocessing of the data in order to get a higher number of spikes per channel, so the results are not usefully comparable; they also did not, as far as can be established, publish an error figure for the standard task.

**Figure 5:**
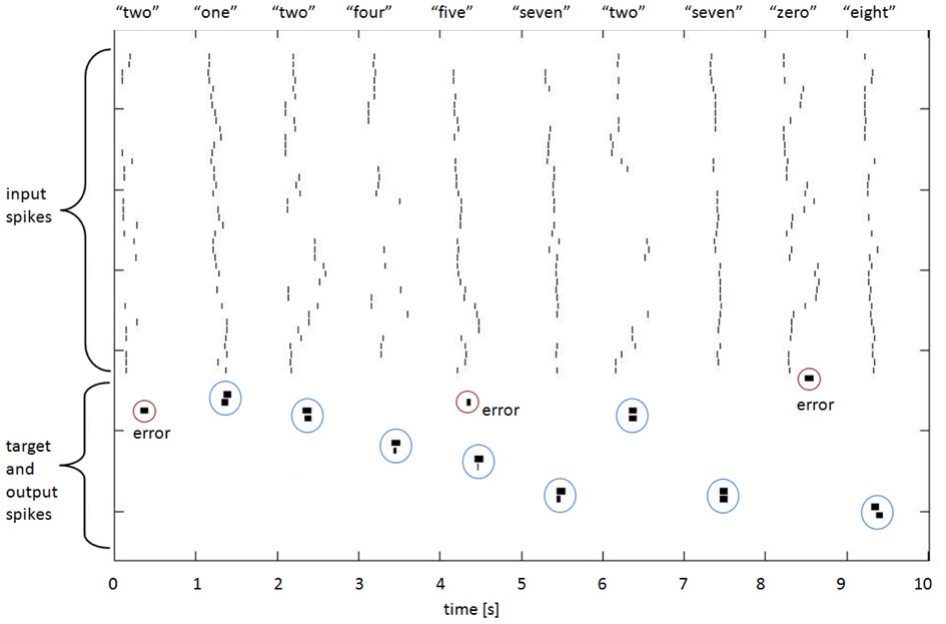
**Spike rasters for 10 spoken digits, showing input, target, and output spikes**. The spike pairs circled with blue dotted lines indicate correct classifications (target and output spikes have been placed together in the raster plot to make visual assessment easier); the other output spikes, circled in red, are errors. The breadth of the target and output spikes—approximately 20 standard spike intervals—is explained in the text below.

Figure 5 illustrates some spike raster patterns for this application.

### Target spike implementation

The *mus silicium* competition data set illustrates an interesting question for synthesis of networks with spiking output: what and when should the output be? If we adhere to the spiking paradigm, then the output should be a spike, but at what time, relative to the input spike set? The TI46 digits are nominally situated in 1-s-long windows, but the variations in spike onset and offset times show that this is by no means a consistent or reliable centering. Nonetheless, for each training exemplar we established at what time the last input spike in that exemplar occurred. We then used that time as the time when the output spike should occur. A glance at Figure 5 shows that when this is applied to the testing patterns, the “target” spike has often commenced before all the input spikes have occurred, which is obviously sub-optimal from a detection perspective (this is because in many of the test cases, the utterances are longer than in the exemplar; one might reasonably expect 50% of them to be so). We made a poor compromise in this case, by spreading the energy of the output spike over 200 ms (hence the visible length in Figure 5), so that there was in effect a lengthy output or target window during which the spike would occur. A moment's thought by the reader will suggest several different and possibly better ways in which this might be done; for example, the output spike could be delayed by some interval that would guarantee that it did not occur before the last input spike, or the target spike could have a trapezoidal spreading of its amplitude to indicate a probabilistic nature. Nonetheless, the network shows useful results with this method, and further research will no doubt improve the performance.

As discussed previously, increasing the amplitude or length of the target signals improves the quality of the training, so using an extended length target pulse as has been done here, is helpful in this regard.

### Errors and capacity

Whilst the SKIM method manages to avoid the large number of spiking neurons used in NEF synthesis, it might be argued that the number of synapses is still unrealistically large in comparison with the complexity of the problem, and that we have replaced the profligate use of spiking neurons with a profligate use of synapses. Current estimates suggest there are on average 7000 synapses per cortical neuron in the adult human brain (Drachman, 2004), so it is not immediately obvious what a correct proportion of synapses might be. We note that biological and computational evidence supports ongoing synaptic pruning as critical in brain function (Paolicelli et al., 2011) and dynamic network optimization (Chechik et al., 1999), so we present here some strategies for reducing synaptic numbers by strategic pruning.

**Figure 6:**
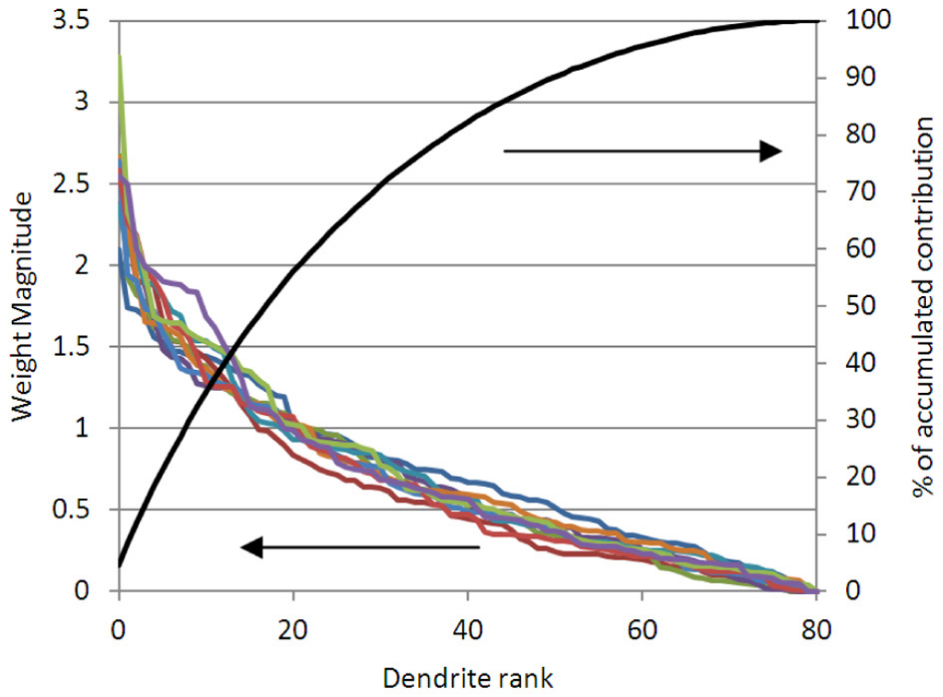
**The magnitude of the solved dendritic weights for 80 dendrites, in 10 different solutions of an example problem like (Figure 3), are shown (left axis)**. It can be seen that in all cases, the 20 largest weighted dendrites are contributing over 50% of the solution magnitude; and that the 40 largest weights are contributing over 80% of the solution (right axis). This suggests that pruning the lowest weighted dendrites will not significantly alter the accuracy of the solution.

In Figure 6 we show the weights for 80 dendrites in the problem of the type shown in Figure 3 (for 10 different iterations, i.e., 10 different non-linear random projections). It can be seen that ~25% of the dendrites are contributing 50% of the weight of the solution; 50% of the dendrites contribute 80% of the weight of the solution. We can follow the physiological practice and prune the dendrites or synapses that are not contributing significantly to the solution. Note that the linear weights must be re-solved after synapses are pruned, or the solution will be non-optimal.

There are numerous strategies by which the weights can be pruned. Two strategies which we have used with success are to over-specify the number of synapses and then prune, in a two-pass process; or to iteratively discard and re-specify synapses. For example, if we desire only 100 synapses, we can synthesize a network with 1000 synapses; train it; discard the 900 synapses which have the lowest dendritic weights associated with them; and then re-solve the network for the 100 synapses which are left. This is the two-pass process. Alternatively, we can specify a network with 100 synapses; train it; discard the 50 synapses with the lowest weights, and generate 50 new random synapses; re-train it; and so on—this is the iterative process. If one prunes synapses which are making a small contribution to the regression solution, then the remaining synapses give a solution which is no longer optimal, so it should be recomputed. The choice of pruning process will depend on the computational power and memory available, but both of these processes produce networks which are more optimal than the first-order network produced by the SKIM method.

## Discussion

The SKIM method offers a simple process for synthesis of spiking neural networks which are sensitive to single and multiple spikes in spatio-temporal patterns. It produces output neurons which may produce a single spike or event, in response to recognized patterns on a multiplicity of input channels. The number of neurons is as sparse as may be required; in the examples presented here, a single input neuron per channel, representing the source of input spikes, and a single output neuron per channel, representing the source of output spikes, has been used. The method makes use of synaptic characteristics to provide both persistence in time, for memory, and the necessary non-linearities to ensure increased dimensionality prior to linear solution. The learning method is by analytical pseudoinverse solution, so has no training parameters, and achieves optimal solution with a single pass of each sample set. We believe that this method offers significant benefits as a basis for the synthesis of all spiking neural networks which perform spatio-temporal pattern recognition and processing.

### Comparison with prior methods

How does SKIM compare to existing models, and in particular those such as LSM and the Tempotron, which are structurally quite similar? There are significant intrinsic differences, upon which we will elaborate below; but the main point of departure is that SKIM is not intended as an explanation or elucidation of a particular neural dynamical system or paradigm, but rather as a method which allows a modeling practitioner to synthesize a neural network, using customized structures and synaptic dynamics, and then to solve the dendritic weights that will give the optimal input-output transfer function. While we consider that its utility may say something about dendritic computation in biology, we consider that it may be useful for modelers who place no value on biological relevance.

In direct comparison with prior methods, we may highlight the following: the most significant difference between SKIM and Liquid State Machines is that SKIM networks are significantly simpler, containing no reservoir of spiking neurons (and in fact having no hidden layer neurons at all). This is not a trivial point, as in the new world of massive neural simulations, the number of spiking neurons required to perform a particular cognitive function is often used as a measure of the success or accuracy of the simulation. While LSM may display complex and rich dynamics as a result of recurrent connections, these come at a price in terms of complexity of implementation and analysis, and in many cases the simpler SKIM network will produce input-output pattern matching of similar utility. We would suggest that a practitioner interested in population dynamics would find more utility in LSM, whereas one interested in spatio-temporal pattern matching with a sparse network, with quick and simple implementation, would find SKIM more useful.

The principle qualitative difference between SKIM and networks with recurrent connections, such as reservoir computing and NEF schemas, is the loss of the possibility of reverberating positive feedback, for use as working or sustained memory. There is no reason that SKIM networks could not be cascaded and connected in feedback; NEF uses the pseudoinverse solution with success in these circumstances. However, we have not yet explored this possibility.

When compared with the Tempotron, the chief attributes of SKIM are the versatility in terms of synaptic structure (the Tempotron is usually described as having exponentially decaying synapses, whereas SKIM can accommodate an extremely wide range of custom synaptic filters); and in having a single-step, analytically calculated optimal solution rather than an iterative learning rule. In the case where SKIM networks are using synaptic functions such as alpha functions or resonances, the peak postsynaptic power from a spike occurs considerably later in time than the spike itself. This has the effect of delaying the spike's contribution in time, and hence acts as a kind of delay line. This has a very useful effect, which is that the soma is no longer performing coincidence detection on the original spikes (which is effectively what the Tempotron does) but on delayed and spread copies, giving it significant versatility.

### Biological plausibility of the SKIM method

Notwithstanding the synaptic pruning possibilities mentioned above, which address possible concerns about synaptic profusion, there are two other areas in which the SKIM method may be considered to be questionably biologically realistic: the use of a pseudoinverse solution, and the use of supervised learning. We have addressed the biological plausibility of linear solutions based on pseudoinverses in a previous report (Tapson and van Schaik, 2013). To summarize, there are incremental pseudoinverse solutions which can be recast in terms of known biological processes such as divisive normalization; and given that this is a convex optimization problem, simpler and nominally more biologically plausible methods such as gradient descent would arrive at the same solution, albeit more slowly. We note also that in contrast to most learning methods, SKIM does not require an error or modification signal to be propagated to the presynaptic neuron, or even the synapse; it is the neuron's own dendritic structure that is modified. The reverse propagation of spikes in dendrites is well-known (Häusser et al., 2000) and could be hypothesized to communicate the modification signal.

Supervised learning is used in SKIM in the standard manner, in the sense of having known target signals to provide an explicit error measure for the weight finding algorithm. The biological plausibility of this feature is arguable, but in the absence of an unsupervised learning methodology, is not likely to be improved upon.

### Time invariance and jitter in the SKIM method

Gütig and Sompolinksy (2009) extended the Tempotron principle by including synaptic shunting conductances, which have the effect of reducing the postsynaptic currents from rapidly incoming spikes (high spike rates), and enhancing the postsynaptic currents from spikes arriving at a low rate, at any particular synapse. This has the effect of producing a high degree of time invariance in Tempotron systems, in terms of robustness of detection of incoming sequences which are subject to time warps.

The SKIM method has three intrinsic features that improve time invariance. The first is that the use of a compressive non-linearity has the effect of reducing the contribution of subsequent spikes arriving within the non-zero envelope of an initial spike's synaptic response. This is similar in effect to synaptic shunting conductance. A second feature is that most realistic synaptic functions have intrinsic spreading as their time constants get longer; for example, the full width at half-height of an alpha function with a time constant of τ = 10 ms is 24 ms; a function with double the time constant (τ = 20 ms) will have double the half-height width (49 ms). If we think of a SKIM network as a multichannel matched filter, then the pass windows (in time domain) for each channel expand linearly in time as the pattern duration gets longer. This gives the network intrinsic robustness to linear time-warped signals. Of course, this does not apply to synaptic kernels where the half-height width does not scale linearly in time. For example, if we wanted a network with high time precision and no robustness to time-warping, we could use synaptic kernels with random explicit time delays and a narrow, fixed width Gaussian function. As the function width narrows toward zero, these networks become very similar to feedforward polychronous neural networks (Izhikevich, 2006); solutions based on dendritic weights lose effectiveness and it becomes necessary to solve for dendritic delays instead. We note that the output or outputs of a feedforward polychronous network, being a spation-temporal pattern of spikes, may be fed back into itself to create a recurrent polychronous network; we have experimented with small networks of this type using the SKIM method to solve the feedforward structure. Finally, the use of synaptic kernels with wide peaks (such as the alpha function) gives the SKIM network some intrinsic robustness to time warping; the alpha function has a full width at half-height that is 244% of the time constant, and this will become even wider after compression by a non-linear function such as *tanh*. Once again, the SKIM method allows the user to choose synaptic features that match particular intuitions, intentions, or known features of the modeled system or the machine learning problem.

### Conflict of interest statement

The authors declare that the research was conducted in the absence of any commercial or financial relationships that could be construed as a potential conflict of interest.
